# Early Biological Valve Failure: Structural Valve Degeneration, Thrombosis, or Endocarditis?

**DOI:** 10.3390/jcm12175740

**Published:** 2023-09-03

**Authors:** Fabio Fazzari, Andrea Baggiano, Laura Fusini, Sarah Ghulam Ali, Paola Gripari, Daniele Junod, Maria Elisabetta Mancini, Riccardo Maragna, Saima Mushtaq, Gianluca Pontone, Mauro Pepi, Manuela Muratori

**Affiliations:** Department of Perioperative Cardiology and Cardiovascular Imaging, Centro Cardiologico Monzino IRCCS, Via Carlo Parea 4, 20138 Milan, Italy; andrea.baggiano@cardiologicomonzino.it (A.B.); laura.fusini@cardiologicomonzino.it (L.F.); sarah.ghulamali@cardiologicomonzino.it (S.G.A.); paola.gripari@cardiologicomonzino.it (P.G.); danielejunod@gmail.com (D.J.); maria.mancini@cardiologicomonzino.it (M.E.M.); riccardo.maragna@cardiologicomonzino.it (R.M.); saima.mushtaq@cardiologicomonzino.it (S.M.); gianluca.pontone@cardiologicomonzino.it (G.P.); mauro.pepi@cardiologicomonzino.it (M.P.); manuela.muratori@cardiologicomonzino.it (M.M.)

**Keywords:** biological heart valves, bioprosthesis, structural valve degeneration, rejection, endocarditis, thrombosis, multimodality imaging, biological valve failure

## Abstract

Biological valve failure (BVF) is an inevitable condition that compromises the durability of biological heart valves (BHVs). It stems from various causes, including rejection, thrombosis, and endocarditis, leading to a critical state of valve dysfunction. Echocardiography, cardiac computed tomography, cardiac magnetic resonance, and nuclear imaging play pivotal roles in the diagnostic multimodality workup of BVF. By providing a comprehensive overview of the pathophysiology of BVF and the diagnostic approaches in different clinical scenarios, this review aims to aid clinicians in their decision-making process. The significance of early detection and appropriate management of BVF cannot be overstated, as these directly impact patients’ prognosis and their overall quality of life. Ensuring timely intervention and tailored treatments will not only improve outcomes but also alleviate the burden of this condition on patients’ life. By prioritizing comprehensive assessments and adopting the latest advancements in diagnostic technology, medical professionals can significantly enhance their ability to manage BVF effectively.

## 1. Introduction

In the last 20 years, the use of mechanical heart valves (MHVs) has declined progressively in favor of the use of biological heart valve (BHV) implantations, which have seen an 80% increase [1]. The excellent hemodynamic profile of BHV, together with an unnecessary lifelong anticoagulation therapy, is responsible for this crucial shift in patients’ treatment. However, despite the technological advances, the durability of BHV is still a major concern. Structural valve degeneration (SVD) is an ineluctable condition that reduces the lifetime of a BHV, leading to a redo valve replacement or a valve-in-valve (VIV) intervention once biological valve failure (BVF) occurs. The mechanisms beyond SVD are various and heterogeneous: dystrophic calcifications, mechanical degeneration, and foreign body rejection. Moreover, the durability of BHVs can be affected by thrombosis, endocarditis, or nonstructural valve dysfunction (N-SVD) caused by intra- and para-valvular regurgitation or prosthesis–patient mismatch (PPM) [2]. An integrated multimodality imaging approach plays a pivotal role in the management of patients with suspected biological valve dysfunction (BVD), from the early stage of diagnosis to the choice of adequate therapy or reintervention. The aim of this review is to provide an overview of the pathophysiology of BVD and the diagnostic multimodality workup in the main different clinical scenarios.

## 2. Durability of Biological Heart Valves

The most commonly implanted BHVs are xenografts made of bovine or porcine pericardia and porcine aortic valves pretreated with chemical agents (glutaraldehyde is the most commonly used) that reduce the immunogenicity of the BHV. Another nonmechanical alternative to BHV is represented by transcatheter heart valves (THVs), which were introduced in 2002. THVs consist of metal stent valves made of cobalt or nitinol with bovine or porcine pericardial leaflets inside [3]. Both surgical heart valves and THVs have limited durability and are prone to SVD. Considering 12 published studies that examined BHV durability over at least 10 years, we observed that SVD occurred in less than 15% of cases, with a need for reintervention that showed a great variability, ranging from 3.3% to 20%, in relation to the model of BHV used [3]. Current evidence on THVs’ longevity is limited to 5–8 years; the NOTION trial, which compared THV (CoreValve) with BHV, showed a similar incidence of BVD but a lower rate of SVD in THV [4].

## 3. Using the Correct Terminology: Biological Valve Dysfunction and Biological Valve Failure

A dysfunctional bioprosthetic valve (BV) is a condition in which the normal function of a BV is altered for structural and nonstructural reasons. Nonstructural BVDs include all conditions that do not implicate a morphological alteration of the components of the valve: prosthesis–patient mismatch, paravalvular regurgitation (with preserved integrity of the BV apparatus), inappropriate positioning of the valve, embolization, or dislocation. Structural BVD is instead determined by pathological changes of the BV: leaflets’ wear and tear, disruption, flail, fibrosis and calcification, strut or stent fracture, or deformation. Structural BVD can also be determined by valve thrombosis or endocarditis. Once structural or nonstructural valve dysfunction occurs, signifying BVD, the process can progress to BVF, a condition characterized by new symptoms’ onset, worsening of the left ventricular function/dimension, or pulmonary hypertension. Finally, BVF can lead to valve reintervention or death.

In the following paragraphs, we focus on organic causes of BVF: SVD, thrombosis, and endocarditis.

## 4. The Three Enemies of Biological Heart Valves: Structural Degeneration, Thrombosis, and Endocarditis

### 4.1. Structural Valve Degeneration (Rejection)

**Figure 1:**
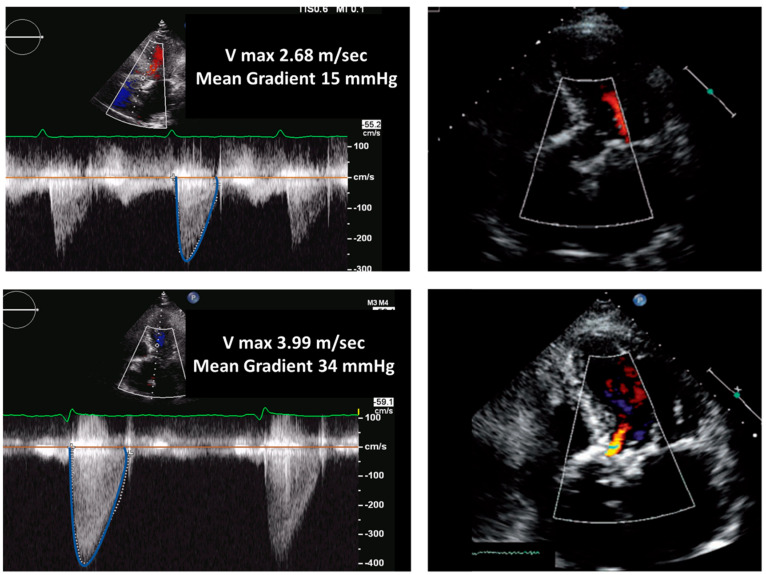
Biological aortic prosthesis at 1 year and 7 years after implantation. Top panels (1-year follow-up): normal gradients and normal morphology of the valve. Bottom panels (7-year follow-up): Significant increase in gradients and thickening of the valve associated with mild regurgitation; these findings are indicative of structural valve degeneration.

**Figure 2:**
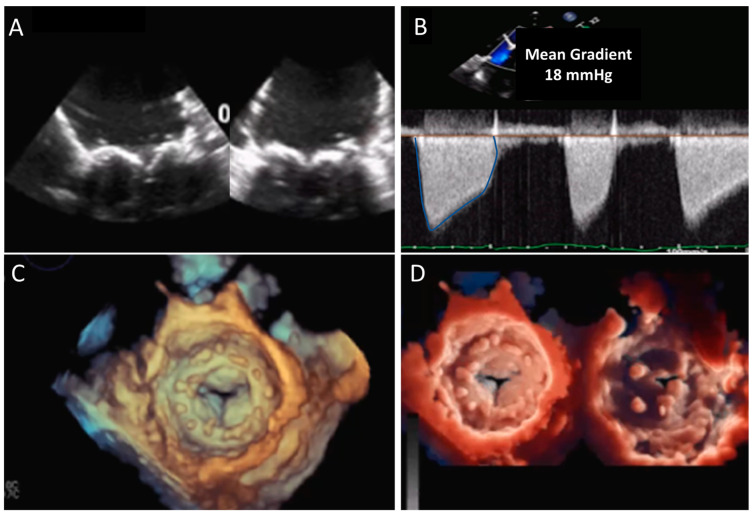
Biological mitral valve prosthesis degeneration. (**A**) TEE mid-esophageal views showing a marked thickening and degeneration of the MV prosthesis leaflets. (**B**)significant increase in the mean diastolic gradient of the prosthesis. (**C**) A 3D TEE surgical view of the MV prosthesis showing a marked reduction in the diastolic opening of the leaflets, as confirmed and further detailed by 3D TEE views from the LV or from the LA with the transillumination modality (**D**). MV, mitral valve; TEE, transesophageal echocardiography.

Native heart valves are a complex multicellular system that is able to self-repair in response to mechanical, hemodynamic, and chemical stressors that occur at every cardiac cycle [5]. On the contrary, BHVs are inert and passively submit to external injuries, leading to a short durability. Calcifications, mechanical degeneration, and immune rejection are the three main mechanisms responsible for SVD. Dystrophic calcifications may occur as a passive or active recipient-related phenomenon. The passive mechanism is related to the internalization of calcium ions (Ca^2+^) into the prosthetic leaflets mediated by chemical pretreatment and precipitation of calcium phosphates (CaPs) into the cells, the collagen, and the elastin fibers. Recipient-related factors (increased calcium intake, severe renal failure, hyperparathyroidism, and inherited deficiency in proteins inhibiting CaPs precipitation) can accelerate calcium deposition together with a cellular-recipient-mediated response through macrophages and red blood cells (RBCs). Macrophages infiltrate the BHV leaflets, leading to apoptosis and CaPs’ deposition, while internalized RBCs cause intra-valvular iron deposition and mineralization. In addition, BHVs are not able to produce an extracellular matrix (ECM) and perform self-repair in response to mechanical stress [5]. Together with the aforementioned processes, the recipient’s immune response makes an important contribution to a further progression of valve dysfunction towards BVF. The native immune system is the main actor of this process through a foreign body reaction [6]. This response starts right after valve implantation, and it is mediated by cytokines and complements cascades’ activation. The result is the adhesion of platelets and activated leukocytes onto the valve’s surface that promotes inflammation, thrombosis, and inflammatory tissue overgrowth. This tissue, also known as pannus, is composed of various cell types: immune cells (macrophages, neutrophils, lymphocytes, and foreign-body giant cells), endothelial cells, and myofibroblasts. SVD and pannus ingrowth may cause stenosis or regurgitation, leading to early BVF. Bovine pericardial valves have a greater propensity to develop stenosis, whereas porcine valves tend to develop leaflet tears with regurgitation. Transthoracic echocardiography (TTE) is key to evaluating the morphological changes of degenerated BV and the hemodynamic consequences of SVD (stenosis or regurgitation). The first target of echocardiographic evaluation must be the search for anatomical changes in the BV’s appearance. BV leaflets’ thickening, calcification, and tearing can be detected by using TTE; however, transesophageal echocardiography (TEE) is more accurate at detecting structural BV changes (Figure 1 and Figure 2). Reduced or excessive leaflet mobility must be described, along with an abnormal color-Doppler pattern, in order to investigate the mechanism of BVD. Flow-dependent and flow-independent Doppler parameters should be used to assess the severity of SVD and BVD. In some cases, a comparison with the baseline echocardiographic study is crucial to determine the presence and the grade of SVD. SVD has been categorized into three stages of severity according to the presence of structural abnormalities and hemodynamic changes assessed by echocardiography [7]; the stages of SVD and the specific echocardiographic parameters are further discussed later in the paper. Both non-contrast and contrast CT scans can easily identify BV leaflets’ calcification, a typical marker of SVD. Furthermore, CT can be used to rule out the presence of thrombus, assess the morphology and size of BV (when unknown), and guide subsequent decision-making about the most appropriate type of valve replacement (surgical vs. transcatheter).

### 4.2. Thrombosis

A meta-analysis published in 2001 that included 5837 patients showed an annual rate of valve thrombosis of about 0.03% (0.3% at 10 years) [8]. However, most of the studies included referred to symptomatic valve thrombosis, masking the real magnitude of the problem. Subclinical valve thrombosis is estimated to be much more common (annual rate of about 0.74%) [9]; however, its clinical relevance is still a matter of debate. The three factors of Virchow’s triad contribute together to the genesis of valve thrombosis (valve surface damage, stasis, and hypercoagulable state). The adsorption of plasma proteins from the surface of the prosthetic valves (in particular, fibrinogen, fibronectin, and the von Willebrand factor) seems to be the key factor that promotes surface damage, platelets’ adhesion, and coagulation cascade activation [10]. These events are triggered by incomplete prosthesis endothelialization, leaflet damage and deterioration, stent fracture, and prosthesis malpositioning. Hemodynamic factors leading to blood stasis can be related both to a low flow state or intrinsic valve characteristics (anatomical position, hemodynamic profiles, malpositioning, and incomplete deployment). Thus, because of the relative right heart slow flow, right-sided valve thrombosis is more frequent than left-sided, and mitral valve thrombosis is more frequent than aortic. Finally, a hypercoagulability state can exaggerate thrombus formation in patients with primitive coagulation disorders or comorbidities such as chronic kidney disease, anemia, smoking, and obesity. As the result of all of these concomitant prolonged injuries, thrombus formation is usually a chronic condition, while fresh thrombi are a rare entity [11].

**Figure 3:**
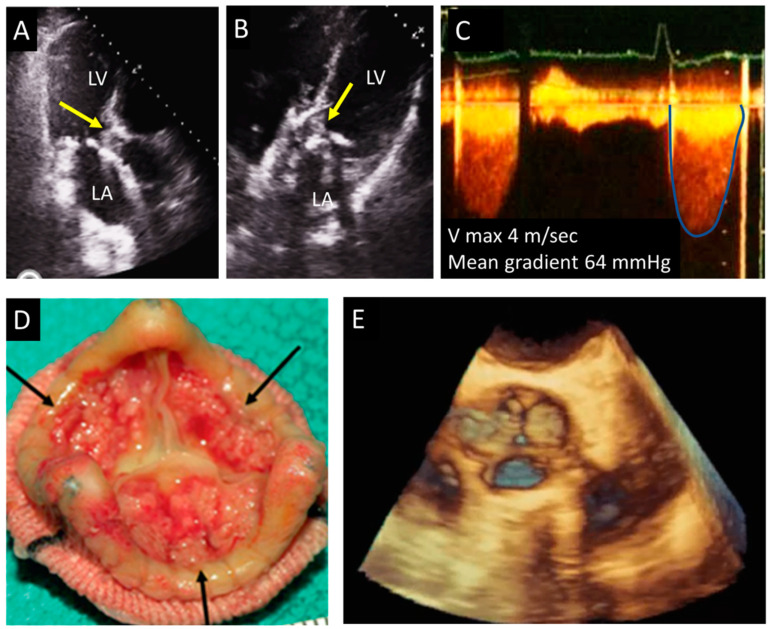
Biological aortic valve thrombosis. (**A**,**B**) Apical long axis and apical 5-chamber adapted views showing thrombosis of the prosthesis (arrows). (**C**) Marked increase of the Doppler systolic aortic gradient. (**D**) Anatomical appearance of the prosthesis (surgical specimen), the arrows indicate the presence of thrombosis of leaflets’ surface. (**E**) 3D TEE imaging of the valve. LA, left atrium; LV, left ventricle; TEE, transesophageal echocardiography.

Valvular thrombosis can be classified into two categories: subclinical thrombosis and clinically significant thrombosis [11,12]. If there is suspicion of leaflet thrombus, such as in the case of TTE, demonstrating an increase in gradient or reduced leaflet motion, or through the occurrence of a clinical event such as thromboembolic events or heart failure, further investigation through contrast-enhanced CT or TEE is necessary to confirm the diagnosis (Figure 3) [13]. Leaflet thrombosis is identified using contrast CT as a hypoattenuated region on leaflets’ surface, called hypoattenuated leaflet thickening (HALT). If a multiphase acquisition is performed, CT can also provide information about leaflet motion; the presence of reduced leaflet motion (RLM) associated with HALT is called hypoattenuation-affecting leaflet motion (HAM), which is synonymous with leaflet thrombus [14]. Subclinical leaflet thrombosis is diagnosed when imaging findings reveal HALT/RLM or HAM, but without significant deterioration of valve hemodynamics or associated symptoms/sequelae. It is worth noting that subclinical thrombosis may spontaneously resolve without any treatment in up to 50% of cases [15]. Currently, there is no evidence to suggest that subclinical leaflet thrombosis significantly impacts clinical outcomes. Therefore, there is no rationale for initiating anticoagulation therapy in the case of an incidental finding of SLT. Anticoagulation therapy with a vitamin K antagonist is generally effective in resolving BVD related to clinical valve thrombosis. However, it is important to note that thrombosis may recur after the completion of treatment. In some cases, valve leaflet fibrosis and calcification may develop, resulting in irreversible structural BVD. This progression can eventually lead to BVF and necessitate reintervention [2].

### 4.3. Endocarditis

**Figure 4:**
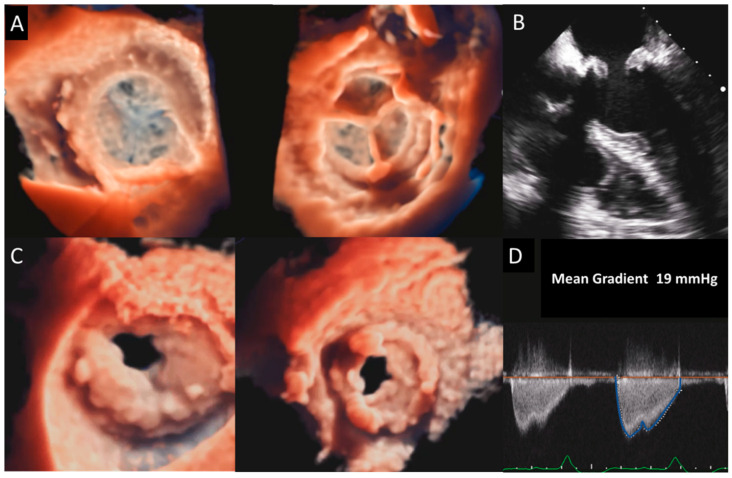
Biological mitral valve thrombosis. (**A**) 3D TEE appearance of the LA and LV of a normal MV prosthesis compared with (**C**) a thrombosis of an MV prosthesis. Thickening and obstruction of the valve are clearly shown by 3D TEE imaging and confirmed by thickening and thrombosis at 2D TEE (**B**) associated with a marked increase in the mean diastolic gradient (**D**). MV, mitral valve; TEE, transesophageal echocardiography.

**Figure 5:**
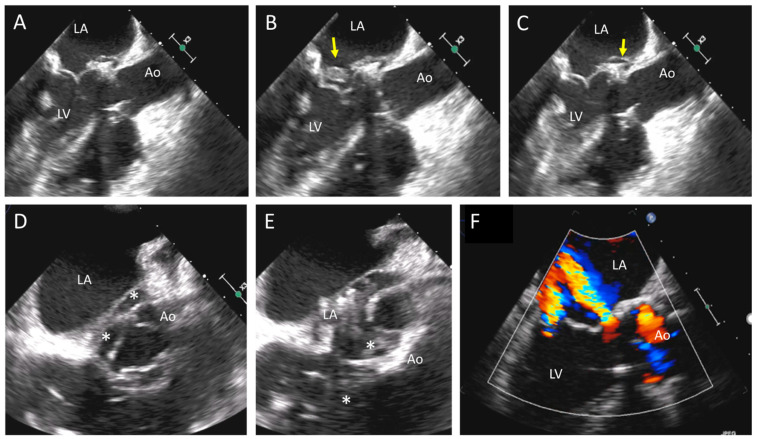
Transesophageal echocardiography of complex endocarditis in a patient with biological mitral and aortic prosthesis. (**A**,**B**) From a mid-esophageal view, there is a normal leaflet appearance (**A**), and in a systolic frame, there is the presence of vegetation (**B**, arrow). (**C**) The arrow indicates an aortic pseudoaneurysm in the mitral–aortic intervalvular region. (**D**,**E**) Short axes of the aortic valve (upper-esophageal view) showing a large abscess of the periaortic tissue (asterisks). (**F**) Color-Doppler also shows a LVOT/left atrial fistula located at the base of the mitral valve. Ao, aorta; LA, left atrium; LV, left ventricle; LVOT, left ventricle outflow tract.

**Figure 6:**
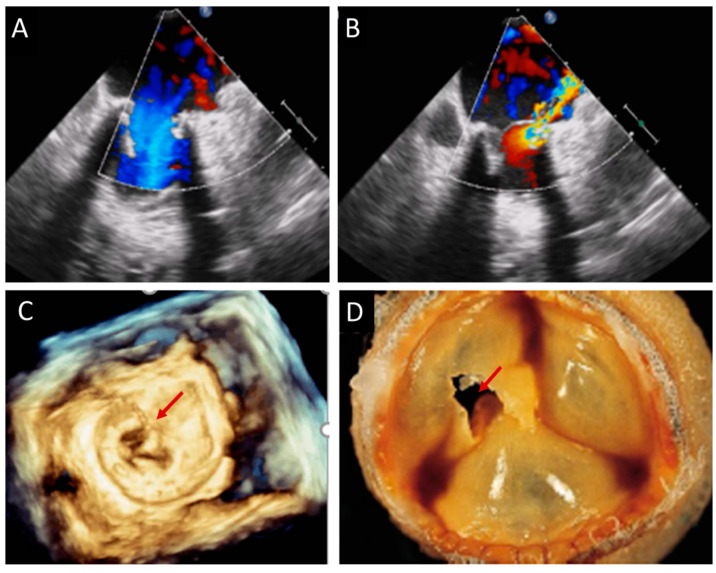
Mitral prothesis endocarditis. (**A**) Diastolic-frame and (**B**) systolic-frame TEE: in these mid-esophageal images, color Doppler shows a pathologic systolic jet at the base of one of the leaflets. (**C**) Perforation (red arrow) and tear of the leaflet are clearly confirmed by surgical view of the valve (3D TEE). (**D**) Example of an anatomical specimen of a perforation (red arrow) of a biological prosthesis. TEE: transesophageal echocardiography.

**Figure 7:**
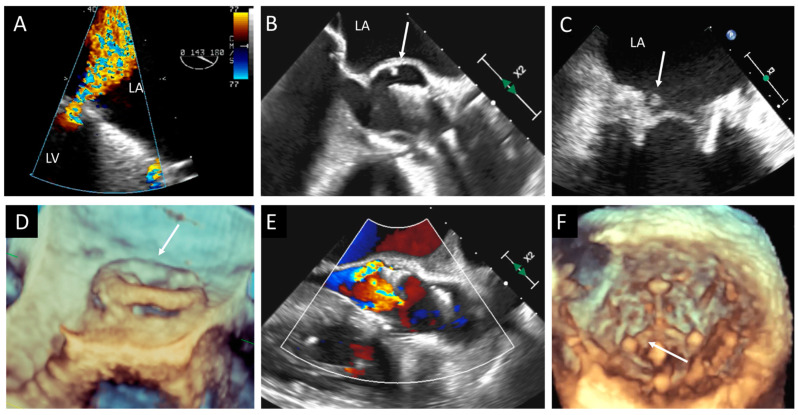
Three cases of prosthetic valve endocarditis: (**A**,**D**) dehiscence and para-prosthetic leak of a mitral valve prosthesis (arrow), (**B**,**E**) pseudoaneurysm (arrow) of an aortic prosthesis, and (**C**,**F**) small vegetation (arrow) in a prosthetic MV visualized in 2D TEE and confirmed in 3D TEE from LV view. LA, left atrium; LV, left ventricle; MV, mitral valve; TEE, transesophageal echocardiography.

The risk of infective endocarditis (IE) is ten times higher in prosthetic heart valves (PVs) than in native heart valves (NVs). Between 10 and 30% of all endocarditis affect PV, with the same incidence among mechanical and biological PV. While staphylococci endocarditis is more frequent during the first year after surgery, other microorganisms seem to cause late-onset endocarditis (mainly streptococci and enterococci) [16]. Early endocarditis usually involves the suture line between the sewing ring and the anulus, causing perivalvular abscess, dehiscence, pseudo-aneurysm, and fistulae. In the late forms, the prosthetic valve leaflets are firstly affected, causing vegetations, cusp rupture, fenestration, or perforation (Figure 4, Figure 5, Figure 6 and Figure 7). A large international multicenter registry showed that prosthetic valve endocarditis (PVE) may also hit THVs, with similar incidence and outcomes of surgical PV [17]. The clinical presentation of PVE may be atypical and challenging, and the Duke criteria have lower accuracy in this setting [18]. TTE has a crucial role when PVE is suspected; however, the presence of shadowing and artifacts may reduce the sensitivity of TTE, and current ESC guidelines suggest to perform TEE in cases of suspected PVE with a negative TTE [19]. Moreover, the use of 3D echocardiography increases the diagnostic power of TEE [20]. In challenging clinical scenarios, 3D TEE allows for a better understanding of paravalvular involvement; the use of multiplanar 3D reconstruction helps in assessing prosthetic dehiscence and valve perforation and in measuring the larger diameter of irregular vegetations [12]. TTE exhibits a sensitivity of 70% for the detection of vegetations and 50% for the detection of abscesses. In contrast, TEE demonstrates a higher sensitivity, nearly 90%, for detecting both types of lesions. Remarkably, the detection rates for both vegetations and abscesses are particularly reduced in individuals with PV compared to other patient populations [21]. In spite of TEE’s heightened sensitivity, TTE remains the initial recommended imaging modality for all patients suspected of presenting with native valve endocarditis (NVE) or PVE. TTE surpasses TEE in its ability to assess left and right ventricular function, as well as to evaluate hemodynamic parameters such as transvalvular gradients, central venous pressure, and pulmonary artery systolic pressure. Additionally, TTE allows for visualization of the anterior aspect of prosthetic aortic valves, which may not be clearly visible with TEE due to acoustic shadowing. It is crucial to highlight that negative findings on either TTE or TEE do not definitively rule out the diagnosis of IE, particularly in the early stages of the disease [19]. During the initial phase, vegetations may be too small to detect, and abscesses may not be visible, as they have not yet begun to form cavities. In such instances, TEE should be repeated after a few days, during which time these lesions are given an opportunity to develop further. Interestingly, a small study demonstrated that Intracardiac Echocardiography (ICE) could be employed in cases of suspected PVE, potentially changing the diagnosis to definite PVE in 50% of cases, without significant additional risks or complications. Therefore, ICE may be utilized when inconclusive results are obtained from TTE and when TEE is contraindicated [22]. IE following transcatheter aortic valve replacement (TAVR) is a rare occurrence; however, its incidence has been increasing due to the growing number of percutaneous valve procedures being performed [17]. Most cases of IE after TAVR occur within the first year following prosthetic insertion, with 2% to 6% of patients developing infections within 5 years [23]. Risk factors for IE after TAVR include valve-in-valve procedures and residual aortic regurgitation, which significantly elevate the risk [16,24]. These factors likely contribute to the formation of jet lesions resulting from turbulence and increased shear stress. Furthermore, structural leaflet changes that are caused by valve manipulation during the procedure increase the risk of IE by inducing local rheologic disturbances. In the presence of bacteremia, these disturbances boost the formation of vegetations. It has been suggested that suboptimal sterility, mainly when TAVR is performed outside of operating and hybrid rooms, can be associated with an increased risk of IE [25]. After TAVR, the most commonly identified microorganisms are Staphylococcus aureus, enterococci, and coagulase-negative staphylococci [25]. Vegetations can be present at the level of prosthetic leaflets, the stent frame, or the mitral valve. In about 20% of patients with IE after TAVR, the infection can spread to the surrounding tissues, causing peri-annular abscess, pseudoaneurysm, and valve detachment. These complications seem to be higher in patients with balloon-expandable valves compared to those with self-expandable valves. IE in this setting is mainly responsible for new onset moderate and severe aortic and mitral regurgitation. Unfortunately, echocardiographic exams may be negative in nearly one-third of patients, suggesting the need for additional imaging methods, such as cardiac contrast CT and/or positron emission tomography (PET). Limited data indicate that performing cardiac CT and/or PET can modify the Duke status in approximately one-third of cases in this context [26]. A CT scan can provide information about local and remote sequalae of IE. Paravalvular abscess is identified as a region with a low-attenuation area with a peripheral enhancing rim, while pseudoaneurysm consists of a perivalvular contrast-material-filled cavity in direct connection with the aortic root or cardiac chamber. The presence of a fistula can be visualized as a contrast-material-filled tract that connects two different cardiac chambers, and it is usually associated with BV dehiscence (the presence of a gap between the annulus and the prosthesis). According to different studies, the sensitivity of CT to detect perivalvular abscess and pseudoaneurysm ranges between 81 and 100%, which is slightly higher than TEE (80–90%) [27].

## 5. How to Distinguish between Valve Degeneration, Thrombosis, and Endocarditis: Integrating Different Imaging Modalities

### 5.1. Echocardiography

TTE is the first-line diagnostic tool for detection of BVD (Figure 1 and Table 1). The current European Society of Cardiology (ESC) guidelines recommend an echocardiographic evaluation after surgical aortic valve replacement (SAVR) or TAVR before discharge, and then a close follow-up between 4 and 6 weeks is needed as a baseline study for further comparisons [28]. Thereafter, the routine follow-up for BHV should be tailored to patient’s and valve’s characteristics; as a general rule, ESC recommends an annual follow-up after the implantation of transcatheter valves, whereas a yearly follow-up should be considered only 5 years after surgical biological valve replacement [29]. In case of signs or symptoms of BVD or clinical suspicion of endocarditis, echocardiography should be promptly performed. The echocardiographic evaluation starts with the anatomical assessment of the prosthesis, followed by color Doppler and hemodynamic evaluation using both flow-dependent and flow-independent Doppler parameters. The key echocardiographic features of SVD are hyperechogenic cusps’ thickening (>2 mm) and hypomobility, calcifications of the cusps and the ring, and tear and flail of the cusps. Pannus can be identified as a hyperechoic fixed layer that is more evident near the ring and the base of the cusps, and it is associated with normal or reduced leaflet motion. Thrombus is usually less echoic (iso or hypoechogenic mass) and adherent to the surface of the cusps, which are thickened and hypomobile. Valve endocarditis can show a wide spectrum of imaging features: presence of vegetations, diffuse or focal leaflet thickening, cusps’ perforation, wear or tear, or cusp avulsion. Moreover, paravalvular complications may be present: abscess, pseudo-aneurysm, fistula, or dehiscence (and, in some cases, valve rocking).

In recent studies [30,31], a proposed categorization of SVD was introduced, encompassing distinct stages of severity, each associated with specific clinical recommendations. Stage 1 of SVD corresponds to early morphological leaflet changes without accompanying hemodynamic consequences. This stage is identified when TTE or TEE reveals abnormal leaflet thickness; increased echogenicity, indicating fibrosis or calcification; or abnormal leaflet mobility, but with normal hemodynamic parameters. Stage 2 SVD refers to morphological abnormalities of valve leaflets, accompanied by hemodynamic dysfunction, such as moderate stenosis or regurgitation. Stage 3 of SVD refers to the development of severe stenosis or regurgitation. A Doppler evaluation is used to confirm severe stenotic hemodynamic parameters or severe prosthetic regurgitation. In the case of Stage 3 SVD, valve reintervention is recommended when symptomatic. SVD stages and related echocardiographic parameters are shown in Table 2 for both aortic and mitral BV. A change in patient clinical status, together with impaired hemodynamic valve function or new-onset regurgitation detected using TTE may call for specific further investigations with either TEE or cardiac CT. In particular, TEE has demonstrated greater accuracy, when compared to TTE, in detecting morphological abnormalities of BV in cases of degeneration, thrombosis, and endocarditis and should be considered mandatory when TTE images are not diagnostic [20,32].

### 5.2. CT

**Figure 8:**
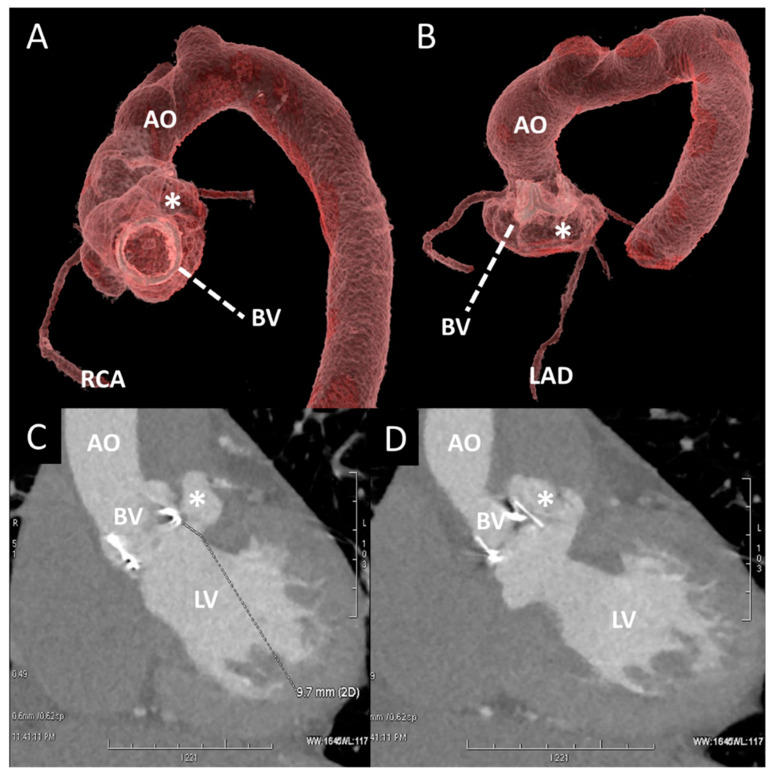
Contrast CT of a pseudoaneurysm (post endocarditis) of aortic root in a patient with aortic BV. (**A**,**B**) A 3D reconstruction of aortic root and thoracic aorta (AO). The asterisk shows the pseudoaneurysm adjacent to the left coronary sinus and the origin of left LAD. (**C**,**D**) Oblique planes of BV and aortic root: in (C), the white line shows the entrance of the pseudoaneurysm (*) right below the BV. BV, bioprosttic valve; LAD, left anterior descending artery; LV, left ventricle; RCA, right coronary artery.

**Figure 9:**
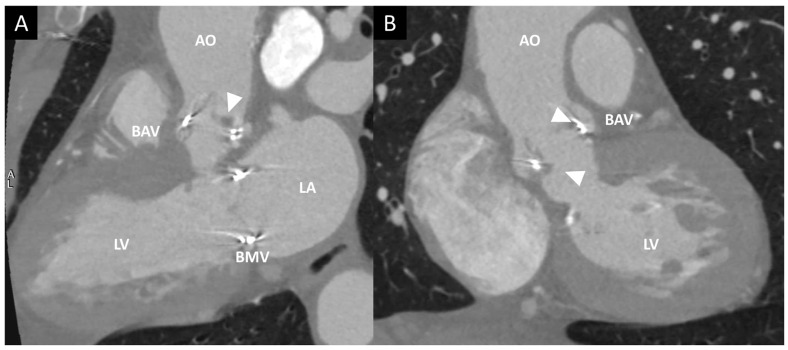
Contrast CT in a patient with endocarditis of a bioprosthetic aortic valve (BAV). (**A**,**B**) Multiplanar reconstruction of LV long axis (**A**) and LV short axis (**B**). Arrowheads show the presence of hypodense masses adherent to BAV leaflets. AO, aorta; BMV, bioprosthetic mitral valve; LA, left atrium; LV, left ventricle.

**Figure 10:**
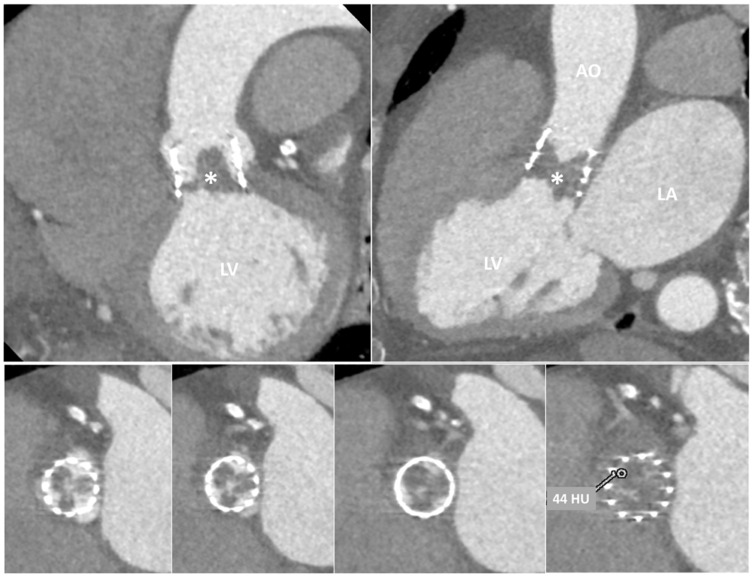
Contrast CT in a patient with transcatheter aortic valve. Upper panels: oblique views of the prosthetic valve (PV); the asterisk indicates the presence of a hypodense mass adherent to prosthetic leaflets. Lower panels: transverse planes of PV showing complete obstruction of the valve; the HU at the level of the mass was 44, indicating the thrombotic nature of the mass. AO, aorta; LA, left atrium; LV, left ventricle.

**Figure 11:**
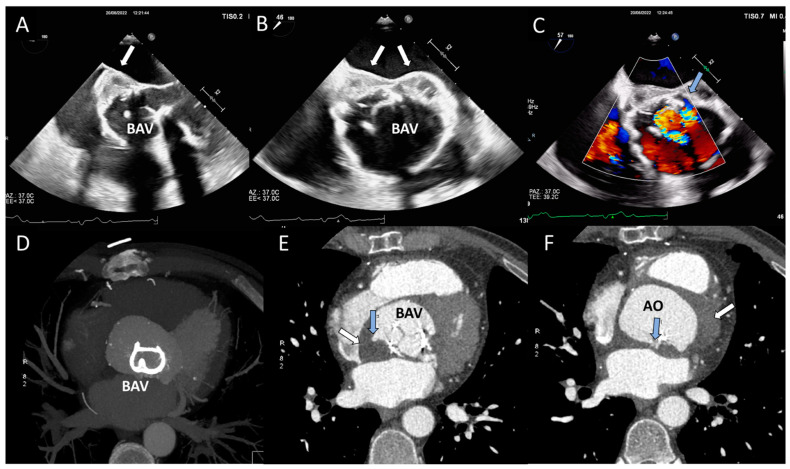
Periprosthetic abscess and detachment in a patient with bioprosthetic aortic valve. (**A**,**B**) Transesophageal echocardiogram in mid-esophageal view showing iso-hypoechoic space posteriorly to the bioprosthetic aortic valve (BAV). (**C**) Transesophageal echocardiogram in mid-esophageal view, with color Doppler showing paravalvular posterior leak and regurgitation (light blue arrow). (**D**) Contrast CT 3D volume rendering: The struts of the BAV are hyperdense and clearly visible. (**E**,**F**) The abscess (white arrow) appears like an isodense region posterior to the BAV; the light blue arrow indicates the presence of multiple leaks with contrast inside.

A poor acoustic window and contraindication to TEE can limit the echocardiographic evaluation in the case of BVD. Cardiac CT (CCT) overcomes these limitations thanks to its higher spatial resolution and its noninvasive nature (Table 1). CCT emerges as the optimal technique for visualizing structural abnormalities and identifying macroscopic calcium deposits. It has the capability to identify leaflet calcification even in the early stages of the disease, even when echocardiography shows normal gradients [33]. Notably, calcification is predominantly observed at the commissures and the basal region of the cusp. While calcium can be detected using non-contrast scans, a threshold of 850 Hounsfield Units (HU) on contrast-enhanced scans has been proposed as a significant cutoff for identifying calcification with an increased risk of paravalvular leak after TAVR [34]. It is important to consider that the structural components of BV, such as the stent frame, can induce beam-hardening artefacts that can mask valve cusps and perivalvular tissue; thus, the accurate knowledge of BV design is crucial to distinguish between artefacts, calcifications, and normal BV components. CCT surpasses other imaging modalities in its ability to characterize thrombus. It effectively visualizes the presence of large, irregular, non-enhancing soft tissue adhering to the leaflets, sewing ring, or both. Furthermore, CCT enables both a quantitative and qualitative assessment of the restricted motion of the leaflets, regardless of the valve’s orientation and position. Distinguishing between thrombus and pannus is of utmost importance to determine the most appropriate treatment approach (Figure 8). A study conducted in 2015 proposed a cutoff value of 145 Hounsfield Units (HU) or higher, which demonstrated a sensitivity of 87.5% and specificity of 95.5% in differentiating pannus from thrombus [35]. This study also provided valuable insights into the optimal treatment strategies and outcomes following thrombolysis for periprosthetic lesions. Thrombus with a CT attenuation of less than 90 HU can be completely dissolved through lysis, whereas the likelihood of complete dissolution diminishes for thrombus, with attenuation values ranging between 90 and 145 HU. Masses exhibiting CT attenuation equal to or greater than 145 HU are indicative of pannus and typically necessitate surgical intervention. SLT, a complication observed following transcatheter aortic valve replacement, has also been subsequently identified in other transcatheter and surgical bioprosthetic valves. Currently, CT is regarded as the gold standard for diagnosing SLT [36]. The characteristic feature of SLT is the presence of hypoattenuating material surrounding the BV, known as HALT. These hypoattenuating lesions are typically observed at the periphery and base of the leaflets, leading to reduced leaflet mobility (RELM). When HALT and RELM are concurrently present, it is referred to as hypoattenuation affecting motion (HAM). Only clinically relevant leaflet thrombosis needs anticoagulation treatment, while STL can be closely monitored without the need for specific therapy [14,37]. In infective endocarditis, CCT demonstrates a high level of accuracy in the assessment of perivalvular complications such as abscesses and aneurysms [27]. Therefore, CCT plays a significant role in preoperatory assessment and timing for reintervention. In a particular study, the inclusion of CCT in the evaluation of individuals with infective endocarditis led to a change in the treatment strategy for 25% of the patients [38]. This imaging modality has the ability to detect life-threatening complications, such as aneurysms and abscesses, which may be overlooked by echocardiography [39]. The CCT findings for PHV endocarditis encompass the visualization of vegetations, aortic wall thickening exceeding 5 mm, perivalvular abscesses, and pseudoaneurysms (Figure 9, Figure 10 and Figure 11). During the CCT, vegetations manifest as irregular, low-attenuation mobile masses adhering to the prosthetic valve leaflet or sewing ring (Figure 9). CCT exhibits remarkable sensitivity in detecting large vegetations (measuring at least 10 mm); however, its capability to assess small vegetations (less than 4 mm) and perforations is limited [18]. Another constraint in evaluating PHV involves streak artifacts caused by the high density of the PHV material. CT also plays a crucial role in the detection of extracardiac abnormalities, such as intra-abdominal lesions (splenic, renal, and hepatic abscesses or infarctions), lesions in the central nervous system, and septic pulmonary emboli.

### 5.3. CMR

Cardiac magnetic resonance imaging (CMR) plays a relatively limited role in the evaluation of BVD (Table 1). CMR has emerged as the reference standard for quantifying chamber size and function and offers a valuable assessment and quantification of valvular heart disease, particularly in cases of valve regurgitation, which is frequently observed in IE or SVD. The unique capabilities of CMR include tissue characterization, such as the detection of myocardial and pericardial inflammation using late gadolinium enhancement (LGE) imaging, as well as parametric mapping techniques. However, it is important to note that despite its excellent spatial resolution, CMR has lower temporal resolution compared to echocardiography, which can limit its ability to detect smaller lesions, such as IE vegetations. Additionally, CMR encounters challenges associated with artifacts stemming from prosthetic valves, device leads, and non-CMR conditional items. Magnetic resonance imaging (MRI) artifacts related to valves are observed as localized regions of signal loss, which can range in severity depending on the quantity and composition of the metallic components involved [40]. A total of 124 patients were examined to evaluate the presence of PHV artifacts, with 115 cases involving biological valves and only 10 cases involving mechanical valves. The presence of signal voids hindered the assessment of the mechanical prosthesis itself, including the valve leaflets. Biological valves equipped with a simple ring did not exhibit any disruptive artifacts, unlike valves with metal struts. Cine sequences can be used to calculate valvular orifice area, while gradients can be estimated through phase-contrast sequences that can help in detecting valve obstruction in the case of SVD, thrombosis, or endocarditis. In the latter case, the role of MRI extends beyond assessing intracardiac manifestations in IE [41]. Brain MRI can provide accurate information regarding cerebrovascular events resulting from vegetation embolization. Magnetic resonance angiography serves as an alternative to CT angiography for evaluating aortic or cerebral mycotic aneurysms, with pooled sensitivities and specificities of 79% and 89%, respectively, as reported in one meta-analysis [42]. It is worth noting that the current guidelines refrain from recommending routine CMR utilization in the evaluation of IE, with the exception of the use of brain MRI to assess cerebral complications and MRI angiography for the evaluation of mycotic aneurysms. Moreover, no study has been reported about the capability of CMR to distinguish between the valvular pannus and thrombus [20]. In general, large thrombi can be identified with CMR by using specific sequences: post-contrast first pass and LGE. The classical appearance of the thrombus is that of a non-enhancing mass during first pass, with a hypointense border and brighter central zone when using LGE sequences. When using LGE sequences with a long inversion time, the thrombus can appear completely dark [43,44].

### 5.4. Nuclear Imaging

^18^F-fluorodeoxyglucose positron emission tomography/CT (FDG PET/CT) and white-blood-cell single-photon emission CT/CT (WBC SPECT/CT) can integrate the initial evaluation of BV diseases (Table 1). FDG is taken up by cells with increased metabolism (neutrophils, lymphocytes, and macrophages) during inflammatory or infective processes. Alternatively, in WBC SPECT/CT, granulocytes are isolated and labeled with ^99m^Tecnetium; once the WBCs are injected, they can then be localized with SPECT/CT. ^18^F-FDG PET offers high sensitivity for the detection of active infection in patients with suspected PVE and inconclusive echocardiography and microbiologic culture results. In a study that compared the two modalities in the same cohort of patients, leukocyte scintigraphy resulted in a higher specificity than ^18^F-FDG PET, suggesting its use in inconclusive ^18^F-FDG PET findings or in the first 2–3 months after cardiac surgery [45]. In the early post-surgery period, FDG PET has a high false-positive rate due to its failure to distinguish between inflammation and infection. Post-pericardiotomy syndrome, the presence of aortic grafts, prosthetic valve thrombosis, and surgical glue are common causes of false positivity when employing FDG PET. Not considering these limitations, when a patient is classified as “possible IE” according to the Duke criteria, the use of PET/CT is able to recategorize the patient in about 90% of PVE cases [46]. Moreover, PET/CT can identify extracardiac complications that are present in about 40% of patients [47]. False-negative cases are represented by small vegetations (<5 mm), recent antibiotic administration, metastatic brain lesions, and high glucose states [48]. Although the main use of ^18^F-FDG PET in the evaluation of prosthetic valve disease is dedicated to PVE, recent data propose its potential use in SVD. ^18^F-fluoride is known to bind to areas of developing microcalcification, an early marker of the macrocalcification that is detectable by CT [49]. In patients with surgically replaced AV with BV, ^18^F-FDG PET was able to predict BV degeneration in ex vivo explanted BV, as confirmed by histology [50]. If confirmed on a large scale, PET-CT could be used to identify patients who are at imminent risk of valve failure, even when CT fails to show valve calcifications. Interestingly, in a multicenter study [51], native valves of patients who underwent both SAVR and TAVI showed evidence of ongoing active disease, as demonstrated by the ^18^F-NaF uptake around the outside of the bioprosthesis during a follow-up of 5 years. In patients with either SAVR or TAVI, the baseline ^18^F-NaF leaflet uptake was predictive of the change in the peak transvalvular velocity on echocardiography. Hybrid modalities are changing our approach to PV failure, moving from an anatomical–functional point of view to the identification of histopathological and metabolic patterns of PV diseases.

### 5.5. Management and Prognosis

**Figure 12:**
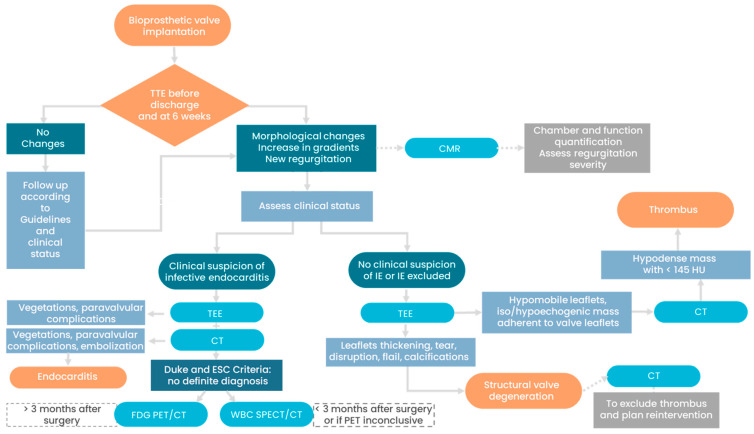
Diagnostic algorithm of bioprosthetic valve dysfunction from a multimodality-imaging perspective. Continuous lines indicate the suggested pathway; dotted lines indicate alternative pathways with lower level of evidence. CMR, cardiac magnetic resonance; CT, computed tomography (both cardiac CT and extracardiac CT); ESC, European Society of Cardiology; FDG PET/CT, ^18^F-fluorodeoxyglucose positron emission tomography/computed tomography; HU, Hounsfield Unit; TEE, transesophageal echocardiography; TTE, transthoracic echocardiography; WBC SPECT/CT: white-blood-cell single-photon emission CT/CT.

A suggested algorithm for the management of bioprosthetic valve dysfunction is provided in Figure 12. TTE is the first-line diagnostic tool, and it should be performed after surgery, before the patient’s discharge. The images and report of the baseline echocardiographic evaluation should be stored using a picture archiving and communication system (PACS) in order to allow for a future comparison during follow-up. TTE must be repeated in the case of a patient’s clinical status change. The echocardiographic examination is usually focused on the research of hemodynamic signs of BV dysfunction (increase in transprosthetic gradients or new regurgitations) and on morphological alterations that can guide the clinician towards a possible diagnosis. The discovery of elevated pulmonary pressure, an increase in ventricular volumes, and a reduction of ventricular systolic function are other important red flags that require further investigations. In the case of clinical suspicion of IE, blood cultures are mandatory, as well as TEE, given the low sensitivity of TTE (17–36%) in this context [32,52]. TTE and CCT can rule out IE in the majority of cases (sensitivity of 82–96% and 88–97%, respectively), while the use of nuclear imaging is restricted to cases with undefined diagnosis according to the Duke and ESC criteria. Moreover, both TTE and CCT can better identify the paravalvular complications of IE and indicate the need for urgent surgery. According to Wang et al., the mortality rate for BVE is higher than that for NVE (23% vs. 16%), and paravalvular abscess is one of the main causes of this increased mortality [53]. SVD usually occurs 7–8 years after surgery, with a higher incidence being seen between 10 and 15 years. Valve thrombosis can be, instead, much more common in the early phases after BV implantation. Non-contrast CCT can show calcifications, which are a typical feature of SVD that is generally absent in the case of valve thrombosis. The presence of a hypodense mass (<145 HU) that is adherent to BV leaflets indicates the presence of thrombosis and the need to start or modify anticoagulation therapy in symptomatic cases. Valve thrombosis is generally resolved after an adequate anticoagulation regimen, but in some cases, it can determine BV deterioration and irreversible SVD. Redo valve surgery is the first option in patients with SVD; however, the majority of studies reported high mortality rates (ranging between 5.8% and 12.8%) for aortic valve surgery [54,55,56]. In the last years, transcatheter VIV procedures have been identified as an alternative in selected high-risk patients. VIV procedures have an overall success rate of 95% for both aortic and mitral BVs, with a 1-year mortality rate of 15% and 20%, respectively (for high-risk patients) [57]. CCT is pivotal for preprocedural planning before surgical and transcatheter treatment of aortic and mitral BVD. If a surgical redo is planned, CCT allows doctors not only to accurately measure the inner diameter of the BV but also to assess the relationship of cardiovascular structures with the sternum, the atherosclerotic burden of the thoracic aorta, and the vascular access routes [58]. In the case of VIV intervention, CCT is even more important than in surgical replacement. The key point is to determine the risk of coronary obstruction before transcatheter aortic VIV. The risk of coronary obstruction following a VIV procedure is higher than for a TAVR of native aortic valve (2.3% vs. 0.66%, respectively) [59]. Patients at high risk of coronary obstruction can be identified using CT by calculating the distance between a virtual model of THV and coronary ostia. Patients with virtual distances <4 mm are considered to be at high risk of procedure-related coronary obstruction. CCT is mandatory in the preprocedural planning before transcatheter mitral VIV; Ge et al. [60] showed that only 36% of patients are suitable for mitral VIV after CCT screening. The most common reasons for exclusion were a large annular size and the risk of left ventricular outflow tract (LVOT) obstruction. LVOT obstruction is associated with a 34.6% mortality rate and a 19.2% rate of conversion to surgery [61]. High-risk features for LVOT obstruction are a small predicted neo-LVOT area (<1.7 cm^2^) [62], short BV-to-interventricular-septum distance [63], acute mitral–aortic angle, interventricular septum hypertrophy, and small hypercontractile left ventricles [64].

## 6. Conclusions

Endocarditis, degeneration, and thrombosis can impact the lifespan of BV and often manifest with similar clinical presentations. The integration of all imaging modalities is crucial for achieving an accurate diagnosis and guiding subsequent patient management. Understanding the strengths and limitations of each modality is essential in identifying the trajectory toward biological valve failure and guiding appropriate therapy and reintervention.

## Figures and Tables

**Table 1:** Typical features of structural valve degeneration, thrombosis, and endocarditis. BV, bioprosthetic valves; CMR: cardiac magnetic resonance; CCT, cardiac computed tomography; FDG PET/CT, ^18^F-fluorodeoxyglucose positron emission tomography/computed tomography; HF, heart failure; HU, Hounsfield Unit; LGE, late gadolinium enhancement; TEE, transesophageal echocardiography; TTE, transthoracic echocardiography; WBC SPECT/CT, white-blood-cell single-photon emission CT/CT.

Typical Features	Structural Valve Degeneration	Thrombosis	Endocarditis
Anatomopathological	Dystrophic calcifications, macrophages, platelet and leukocyte infiltration, and inflammatory tissue overgrowth (pannus).	Fresh or chronic thrombi adherent to leaflets’ surface may be associated with leaflets’ surface damage.	Vegetations adherent to upstream surface of valve leaflets, made of inflammatory cells and microorganisms. Paravalvular disruption.
Clinical	Usually late onset of symptoms (5–10 years after surgery) and slow progression of BV dysfunction at serial monitoring.	Subclinical thrombosis is an incidental finding (spontaneous resolution in 50% of cases). Clinical thrombosis: heart-failure symptoms and thromboembolic events.	Staphylococci endocarditis (first year after surgery); other microorganisms (late onset). Fever and symptoms of infection precede rapid onset of HF symptoms. Thromboembolic events.
TTE, TEE	Leaflet and/or ring thickening with diffuse or focal hyperechogenicity (calcifications). Reduced leaflet mobility. Leaflets’ fenestration, avulsion, or perforation. Stenosis or regurgitation.	Iso-hypoechogenic mass adherent to leaflets and ring, with leaflet thickening. Normal or reduced cusp mobility. More often, stenosis; regurgitation is uncommon.	Vegetations, diffuse or focal leaflets thickening, cusps perforation, wear or tear or cusp avulsion. Vegetation motion independent to cusps motion. Paravalvular complications: abscess, pseudo-aneurysm, fistula or dehiscence (and in some cases valve rocking)
CCT	Pannus (hypodense): HU ≥ 145; semicircular or circular structure located along leaflets’ surface or stent. Hyperdense leaflet thickening with or without calcifications, along with or without reduced mobility.	No calcifications. HU < 145. Hypoattenuated leaflet thickening (HALT), affecting (HAM) or reducing (RLM) leaflet motion. In some cases, large hypoattenuated mass.	Hypoattenuated mass adherent to leaflets or stent. Paravalvular complications: abscess, pseudo-aneurysm, fistula, or dehiscence.
CMR	Limited role in anatomical definition. Quantification of chambers volume and function. Quantification of stenosis and regurgitation (when echo is not feasible).	Non-enhancing mass during first pass with hypointense border and brighter central zone at LGE sequences.	Small and highly mobile vegetations are not visible. Main use to detect extracardiac manifestations (cerebral and aortic).
PET/CT or SPECT/CT	No BV or pannus ^18^FDG uptake. Possible ^18^F Na uptake of BV leaflets.	Possible ^18^F Na uptake of BV leaflets.	Increased ^18^FDG uptake of BV leaflets, paravalvular regions, and metastatic foci.

**Table 2:** Stages of structural valve degeneration. The impairment of prosthetic aortic and mitral valves hemodynamic is associated with abnormal echocardiographic parameters for each stage. DVI for aortic prosthesis is calculated as LVOT VTI/aortic VTI; DVI for mitral prosthesis is calculated as mitral VTI/LVOT VTI. AT, acceleration time; DVI, Doppler velocity index; EOA, effective orifice area; ET, ejection time; MVA, mitral valve area; LVOT, left ventricular outflow tract; V max, maximum velocity; SVD, structural valve degeneration; VTI, velocity time integral.

SVD Stages	Aortic Valve Bioprosthesis	Mitral Valve Bioprosthesis
Stage 1	Morphological valve deterioration without hemodynamic impairment.	Morphological valve deterioration without hemodynamic impairment.
	Vmax: <3 m/s; Mean gradient < 20 mmHg, with an increase in mean gradient during follow-up < 10 mmHg; DVI > 0.35; AT < 100 ms; AT/ET < 0.32; EOA > 1.2 cm^2^ for BSA < 1.6 m^2^; EOA > 1 cm^2^ for BSA < 1.6 m^2^.	Mean gradient < 5 mmHg; DVI < 0.4; MVA > 1.5 cm^2^.
Stage 2	Morphological valve deterioration plus one of the following:	Morphological valve deterioration plus one of the following:
	Vmax: 3–4 m/s; Mean gradient: 20–40 mmHg, with an increase in mean gradient during follow-up between 10 and 20 mmHg; DVI between 0.25 and 0.35; AT between 80 and 100 ms; AT/ET between 0.32 and 0.37; EOA between 1 and 1.2 cm^2^ for BSA < 1.6 m^2^; EOA between 0.8 and 1.1 cm^2^ for BSA > 1.6 m^2^; Moderate regurgitation.	Increase in DVI ≥ 0.4 or ≥20% resulting in DVI ≥ 2.2; Decrease in MVA ≥ 0.5 cm^2^ or ≥25% resulting in MVA < 1.5 cm^2^, compared with echocardiographic assessment performed post-surgery; Mean gradient > 5 mmHg; Moderate regurgitation.
Stage 3	Morphological valve deterioration plus one of the following:	Morphological valve deterioration plus one of the following:
	Vmax > 4 m/s; Mean gradient > 40 mmHg, with an increase in mean gradient > 20 mmHg during follow-up; AT > 100 ms; AT/ET > 0.37; EOA < 1 cm^2^ for BSA < 1.6 m^2^; EOA < 0.8 cm^2^ for BSA > 1.6 m^2^. Severe regurgitation	Increase in DVI ≥ 0.8 or ≥ 40% resulting in DVI ≥ 2.5; Decrease in MVA ≥ 1.0 cm^2^ or ≥50% resulting in MVA < 1.0 cm^2^, compared to echocardiographic assessment performed post-surgery; Mean gradient > 10 mm Hg during follow-up; Severe regurgitation.

## Data Availability

Not applicable.
